# In Vivo Mitochondrial Function in Idiopathic and Genetic Parkinson’s Disease

**DOI:** 10.3390/metabo10010019

**Published:** 2019-12-28

**Authors:** Gabriele Dossi, Letizia Squarcina, Mario Rango

**Affiliations:** Excellence Center for advanced MR techniques and Neurology unit, Fondazione IRCCS Cà Granda Maggiore Policlinico Hospital, Via Sforza 35, 20122 Milan, Italy; gabridossi@gmail.com (G.D.); letizia.squarcina@gmail.com (L.S.)

**Keywords:** brain energetics, parkinson’s disease, PINK1 mutation, mitochondria, ^31^P-MRS, phosphorus

## Abstract

Parkinson’s disease (PD) is associated with brain mitochondrial dysfunction. High-energy phosphates (HEPs), which rely on mitochondrial functioning, may be considered potential biomarkers for PD. Phosphorus magnetic resonance spectroscopy (^31^P-MRS) is a suitable tool to explore in vivo cerebral energetics. We considered 10 ^31^P-MRS studies in order to highlight the main findings about brain energetic compounds in patients affected by idiopathic PD and genetic PD. The studies investigated several brain areas such as frontal lobes, occipital lobes, temporoparietal cortex, visual cortex, midbrain, and basal ganglia. Resting-state studies reported contrasting results showing decreased as well as normal or increased HEPs levels in PD patients. Functional studies revealed abnormal PCr + βATP levels in PD subjects during the recovery phase and abnormal values at rest, during activation and recovery in one PD subject with PINK1 gene mutation suggesting that mitochondrial machinery is more impaired in PD patients with PINK1 gene mutation. PD is characterized by energetics impairment both in idiopathic PD as well as in genetic PD, suggesting that mitochondrial dysfunction underlies the disease. Studies are still sparse and sometimes contrasting, maybe due to different methodological approaches. Further studies are needed to better assess the role of mitochondria in the PD development.

## 1. Introduction

Parkinson’s disease (PD) is a common neurodegenerative disorder with several layers of complexity. PD mainly affects the dopaminergic neurons of Substantia Nigra Pars Compacta (SNPC) causing their death and, as a result, an overall impairment within the basal ganglia leading to the typical motor symptoms of PD such as tremors and rigidity [1]. Also, PD is characterized by non-motor symptoms such as psychiatric disorders, gastrointestinal issues, sexual concerns, and cognitive decline [1]. People usually develop idiopathic PD around the age of 60 even though PD can occur before as early onset Parkinson’s disease (EOPD, age of onset < 45 years.) [2]. In turn, EOPD is distinguished by a phenotypic variability between subjects with genetic mutations such as PTEN-induced kinase mutation 1 (PINK1), a gene that encodes serine-threonine kinase, and subjects without mutations. [3].

Several lines of research suggest that mitochondrial dysfunction plays a critical role in the development of both idiopathic and familial PD [4,5]. Indeed, various studies reported impairments in the respiratory chain due to environmental toxins (e.g., rotenone) that are powerful inhibitors of mitochondrial Complex I while post-mortem studies found widespread impairments in Complex I and Complex II of the respiratory chain in SNPC, hippocampus, putamen, and pedunculopontine nucleus of PD patients [4,6,7]. All these alterations render neurons more vulnerable to apoptosis and excitoxicity and, as a result, they cause dysfunction and neural death in PD. Furthermore, several findings highlighted the link between mitochondrial dysfunction and alpha-synuclein aggregation underlining that oxidative damage seems to impair the regular degradation process of protein leading to the Lewy bodies genesis [8,9].

In the last year, genetic studies are suppling further evidence on the critical role played by mitochondria in the pathogenesis of PD [4,5]. Among the many pathogenic gene mutations, autosomal recessive *Parkin* and *PINK1* mutations seem to be strictly linked to mitochondrial dysfunctions [10]. *Parkin’s* main role is to regulate mitochondrial mitophagy and biogenesis and its mutations are the first cause of autosomal recessive PD [11]. *PINK1* gene mutations, which are the second most common cause of autosomal recessive EOPD, alter mitochondrial homeostasis and impair mitophagy of damaged mitochondria [10,11,12,13,14]. Furthermore, PINK1 mutations cause Ca^2+^ overload in mitochondria and impairments in Complex I and Complex III of the respiratory chain [15,16]. As well, GBA gene mutation is another mutation linked to PD development [5]. Whereas homozygous GBA mutation cause Gaucher disease (GD), GBA heterozygosity is a potential risk factor for developing PD with an earlier age of onset and a major risk to develop dementia compared to idiopathic PD [17]. Moreover, recent studies showed that GBA heterozygous mutation is linked with dysfunction in mitophagy [18,19].

All these findings suggest that mitochondrial impairments, due either to environmental or genetic causes, are strictly related to several neurodegenerative mechanisms such as oxidative stress and energetics impairments that are hallmarks of PD pathogenesis.

The study of mitochondrial function may result in being critical not only for investigating PD pathogenesis but also for developing prevention strategies, new treatments, and more focused therapies even at the early stage of the disease.

Mitochondrial functioning can be investigated by magnetic resonance spectroscopy (MRS) [20]. Indeed, in MRS, magnetically active nuclei (e.g., ^1^H, ^31^P) are polarized by a strong external magnetic field and, successively, they are excited, within a volume of interest, by a broadband radio frequency (RF) pulse whose frequency is wide enough to cover the range of frequency of interest. After recording and mathematically processing the response of the nuclear spins to the RF pulse, it is possible to yield a spectrum containing peaks at specific resonant frequencies called “chemical shifts” along the x-axis (expressed in parts per million (ppm)) whereas corresponding peak amplitudes are reported on the y-axis. Since each metabolite has a specific chemical shift, it is possible to recognize a specific metabolite along the frequency (x-axis) and its concentration along the peak amplitudes (y-axis) reported in the spectrum [20].

Phosphorus magnetic resonance spectroscopy (^31^P-MRS) is a non-invasive technique that allows one to study brain energetics and mitochondrial function in vivo revealing itself as a suitable tool to measure phosphorus compounds linked to brain energy metabolism such as free phosphate (Pi), phosphomonoester (PME), phosphodiester (PDE), and the high-energy phosphates (HEPs), namely adenosine triphosphate (ATP) and Phosphocreatine (PCr) [21]. These compounds are markers of the oxidative phosphorylation and mitochondrial function. Indeed, βATP can be considered as a marker of total ATP concentration, PCr the most important short-term energy reserve, and ADP the major regulator of mitochondrial respiration [22,23]. PCr is a high energy source that supplies a phosphate group for ATP production from ADP via the creatine phosphokinase-catalyzed in case of increased demand for ATP due to decreased oxygen availability, enhanced energy request, or not sufficient ATP production via oxidative phosphorylation. ^31^P-MRS reflects this process reporting decreased levels of PCr, increased levels of Pi, and steady ATP levels [20]. Moreover, ^31^P-MRS can be used to measure the intracellular pH since the chemical shift of Pi is pH dependent and to investigate the precursor of neuronal membrane lipids (e.g., phosphocholine (PCho) and phosphoethanolamine (Peth)), membrane lipid catabolites such as glycerophosphoethanolamine (GPE) and glycerophosphocholine (GPC) in order to obtain information about the membrane integrity as well as other parameters [20,22]. In light of this, potential mitochondrial dysfunction and impaired energy metabolism may be considered important biomarkers of PD and they can be detected via ^31^P-MRS.

In this context, this review aims at summarizing all ^31^P-MRS studies that investigated mitochondrial and energetics function in idiopathic PD and PD linked to gene mutations.

We carried out a bibliographic search in PubMed and Scopus using “*Parkinson AND Phosphorus*” as keywords. No time restrictions were used and we selected studies until October 2019. We excluded studies that: (a) Employed neuroimaging techniques other than ^31^P-MRS, including studies employing only proton magnetic resonance (1H-MRS); (b) were not in humans; (c) explored PD in subjects with psychiatric issues and/or dementia. More generally, in this review, we discuss results and data obtained only by ^31^P-MRS investigation.

Inclusion criteria were met by 10 studies whose methods and main results are summarized in Table 1. 

Eight studies were performed using at rest ^31^P-MRS technique while two studies used a functional approach. PD diagnosis and symptoms severity were mostly assessed with the UK Parkinson’s Disease Society Brain Bank criteria, the Hoehn and Yahr scale, and the Unified Parkinson’s Disease Rating Scale (UPDRS). Specifically, one study compared idiopathic PD with multiple system Atrophy (SMA), one study focused on PD with heterozygous glucocerebrosidase mutations (GBA-PD) and two studies investigated the PINK1 mutation related to EOPD. The rest of the studies were on idiopathic PD.

For the sake of clarity, the results obtained by resting-state and functional ^31^P-MRS will be reported and discussed in different sections. 

## 2. Resting-State ^31^P-MRS 

The aim of this review was to reconsider the ^31^P-MRS role for the study of mitochondrial function and energetics in patients affected by PD. In light of this, ^31^P-MRS is a powerful tool to investigate in vivo mitochondrial. However, despite its potential, its practice is still limited.

The first study was performed by Montagna and colleagues who studied 10 PD patients focusing on frontal lobes and basal ganglia [28]. The authors reported similar brain levels of PCr, ADP, and pH between PD and HC, but noticed increased Pi values in the PD group and consequently a low level of readily available free energy in the nerve cells, expressed by the phosphorylation potential (PP = [ATP/ADP]*[Pi]). Likewise, Barbiroli and colleagues, working on a sample of PD and MSA patients, found higher values of Pi in the occipital cortex of PD compared to HC and greater values of PCr in PD group if compared to MSA patients [24]. Similarly, Hu and associates observed increased values in the mean Pi/βATP ratio in the bilateral temporoparietal cortex of PD patients, with a specific increment of Pi values in the right temporoparietal cortex [27]. Significant decreases of βATP in thalamus, globus pallidus, and brainstem (included SNPC) were noticed linked to increased values of PME/ βATP, PDE/βATP, PCr/βATP, and Pi/βATP ratios. Interestingly, the authors administered cognitive tests to the PD patients and they found important correlations between Pi/βATP ratio in the temporoparietal cortex and reduced performances in neuropsychological tests assessing cognitive and language function. Overall, all these studies found enhanced levels of Pi in PD patients in several brain regions such as frontal lobes, occipital lobes, and temporoparietal cortex suggesting that mitochondrial oxidative phosphorylation is deranged in PD causing abnormal cell ion environment and ion traffic [32]. Oxidative phosphorylation impairment was also suggested by several studies that found enhanced Pi/βATP ratio and significant reduction of N-Acetylaspartic acid (NAA) levels in the temporoparietal cortex as well as reduced ATP and PCr levels in thalamus, basal ganglia, and midbrain [26,27,33]. NAA is synthetized only by mitochondria and it is present only in the neural cells whereas ATP levels begin to fall when the PCr pool is reduced by 50%. All these findings provide robust evidence of mitochondrial incapacity in maintaining adequate levels of ATP and coping with high energy demands. 

Interestingly, two studies tried to find potential biomarkers for early PD diagnoses but with contrasting results. Hattingen and colleagues found reduced ATP values in midbrain and putamen of PD patients both at early and advanced stage of the disease whereas reduced PCr values were reported in bilateral putamen independently from duration of the disease. PCr + βATP compounds resulted in decreases in all contralateral hemispheres investigated; conversely, in the ipsilateral hemisphere, the decrement was only in the putamen [26]. On the other hand, Weiduschat and colleagues did not find any difference in metabolites amount between PD patients at early stage and HC [31]. This dissimilarity could be ascribed to methodologic differences or to clinical heterogeneity of the samples. Nevertheless, since early diagnosis is crucial for better therapeutic strategies, this aspect is worth to be examined in depth.

Sex differences seem to be a critical factor in the development of several neurodegenerative diseases such as Alzheimer’s Disease, Huntington’s Disease, and PD. PD affects more men than women and the male sex resulted to be one of the most remarkable PD risk factors [34]. In light of this, Weiduschat and colleagues explored mitochondrial function in PD in order to find potential differences regarding sex and they found higher ATP values in the striatum of women patients as well as in the temporoparietal grey matter contralateral to the more affected body size. On the other hand, PCr and ADP levels were higher in bilateral striatum and bilateral grey matter [30]. This could be due to the estrogen long-term effect that is known to enhance oxidative phosphorylation and to reduce ATPase activity suggesting that sexual dimorphism could underlie metabolic differences other than clinical phenotype in PD [35,36]. However, since this is the only MRS study on metabolic sex differences in PD, further studies are needed.

Even though, in the last year genetic, studies associated with PD are increasing, to our knowledge, only two resting-state ^31^P-MRS studies investigated mitochondrial functioning in individuals with gene mutation linked to PD. Specifically, Hilker and colleagues focused on 11 members of a German family with hereditary EOPD due to PINK1 mutation [22]. Subjects with heterozygous PINK1 mutation with mild clinical manifestations (PINK1-) did not show energetic differences compared to HC, whereas homozygous PINK1 mutation carriers with clinical manifestations (PINK1+) revealed both higher putaminal βATP and PCr values than HC. Moreover, PINK1 + showed levels of putaminal GPE and GPC above the normal range. These unexpected results in homozygous PINK1+ patients were explained with an impaired oxidative phosphorylation compensated by an enhanced ATP production via glycolysis maybe due to astrocytes compensatory activity [37,38]. Furthermore, the authors reported an inverse correlation between high levels of GPC and low levels of dopamine uptake in PINK1+ patients. Notably, abnormal GPC levels were also reported in other progressive neurodegenerative diseases such as Alzheimer’s Disease (AD) suggesting that they can be considered marker of neural membrane deterioration due to oxidative stress [39].

Brockmann and colleagues carried out a study on neuronal integrity, energy metabolism, and membrane phospholipid metabolism in a sample of GBA-PD patients. Limited to energy metabolism in the putamen and in the midbrain, the authors did not find differences in the concentrations of HEP and low-energy phosphates (LEP) except for a decreased amount of creatine (Cr) in the GBA-PD group [25]. These results are in contrast with the other ^31^P-MRS studies mentioned above. Conversely, the phospholipidic membrane catabolite GPE level, which was normal in PD, was more concentrated in the GBA-PD patients. Although the role of impaired membranes in GBA-PD is still not clear, it has been hypothesized that it is strictly linked to abnormal α-synuclein deposition in the brain [40,41]. Similar impairments in the phospholipidic membrane were also noticed in PINK1+ patients even if those patients showed energetics impairments. This suggests that there might be a sort of connection between membrane deterioration and genetic mutations linked to PD and a potential link between phospholipidic membranes impairments and mitochondria function [42,43,44]. In this respect, further analyses are needed.

Taking all this into account, we believe that all these studies should be considered in the light of some limitations. Indeed, three studies measured intracellular energy using ratios (e.g., PCr/ATP and PCr/Pi) and the other studies calculated PCr + ATP content using the 1H-MRS noise as reference, thus not allowing full quantitation. 

## 3. Functional ^31^P-MRS 

If resting-state ^31^P-MRS studies are sparse, functional ^31^P-MRS studies are even fewer. To our knowledge, Rango and colleagues are the only who performed MRS studies on PD patients under dynamic conditions in order to show directly mitochondrial impairment in the brain of PD patients. Although a complete investigation on mitochondrial function requires the study of brain energetics at rest and during activation and recovery, MRS dynamic studies are still sparse because there are methodological difficulties and temporal and spatial limitations [45]. In order to fix these problems, Rango and associates developed a method to study mitochondrial function in the brain visual cortex during a short activation with a spatial resolution of 10 cm^3^ [45]. This innovative method resulted very useful for the investigation of energetics in PD for several reasons: Firstly, visual stimuli could be easily graded; secondly, visual cortex has high oxidative metabolism and high level of oxidative phosphorylation enzymes [46]. Finally, there is lower risk of the occurrence of confounding effects due to L-dopa or suffering brain cells in visual cortex as, conversely, it happens in striatum and midbrain areas [45]. Furthermore, as opposed to the other mentioned studies, the authors used the reference compound (mmol) that allows one to provide a full quantitation of the HEP content. [23,29,45].

Applying this method on a sample of 10 idiopathic PD patients, Rango and colleagues found abnormal values in the PD group only during recovery phase reduction, reporting a decrement in quantitative PCr + βATP levels [23]. This suggests that, since during activation the energy request is low and mainly sustained by anaerobic glycolysis whereas recovery requires increased energy via oxidative phosphorylation, the brain of PD patients is not able to sustain high energy demands. Interestingly, similar results were obtained on a sample of patients with mitochondrial disease without CNS involvement [47]. All these findings indicate that mitochondrial impairment in PD is not linked to the oxidative damage of dopaminergic metabolism, since the visual cortex is not implied in such metabolism, but it is an additional factor that worsens the mitochondrial function of striatum and substantia nigra neurons. Moreover, the authors replicated the study on a sample of one patient affected by EOPD with heterozygous PINK1 mutation and 10 idiopathic PD patients [29]. The PINK1 patient showed reduced level of PCr + βATP at rest, during activation, and during recovery if compared to HC whereas PD patients, similarly to the previous study, showed impairments only during the recovery phase. Furthermore, no compensatory effects due to glycolysis were reported. All these results are in contrast with the study carried out by Hilker and associates [22]: These discrepancies may be explained by different methodological approach and by the small sample investigated. Altogether, the current results showed that brain energetics are impaired in PD, specifically in the EOPD variant linked to PINK1 mutation, where mitochondrial machinery is more heavily affected.

## 4. Limitations

This review should be considered in the light of some limitations. First, brain areas investigated by ^31^P-MRS and methodological approach were not the same for every study, which may explain some heterogeneity in the results. Second, the samples considered in the reported studies were small and included patients with different age, sex, intensity symptoms, and duration of the disease. Particularly, studies investigating PINK1 mutation reported either only one patient or a familial case. Third, the use of medication should be considered as a confounding factor even if no significant relations were found with energetics and metabolites compound since the samples were not homogenous regarding the kind of treatment. Finally, it is important to underline that this review considers study in a range of 22 years so technological innovations need to be taken into account in relation to the results.

## 5. Conclusion and Future Perspectives

In conclusion, mitochondrial impairments in the oxidative phosphorylation process could be potential biomarkers for both idiopathic PD and familial PD. However, the causes of these dysfunctions are still not clear and, importantly, are not limited to brain areas more affected by PD. The investigation of energetic metabolites by improving ^31^P-MRS technique and, specifically, using functional approaches may facilitate not only the knowledge about the role of mitochondrial function in PD pathogenesis and its subclinical variants, but also would ameliorate the development of new therapeutic strategies. Even though ^31^P-MRS potential is still not fully exploited, this technique may be critical in the study of PD, specifically in the early stage of the disease or in EOPD when the symptomatology is more ambiguous and treatments would result more useful. For all these reasons, we firmly believe that ^31^P-MRS studies should be improved. Particularly, we suggest to enhance functional studies in order to better understand the mitochondrial functioning, even in brain areas more impaired by PD than visual cortex. Then, we recommend to increase functional MRS studies in subjects with gene mutations linked with PD that impair mitochondrial function such as *Parkin* and *PINK1*. Finally, we think that a combined approach of genetics, MRS, and MRI techniques should be better exploited in order to find further potential biomarkers of mitochondrial dysfunction. For instance, brain temperature may reveal a biomarker for PD. Indeed, proton-MRS (^1^H-MRS) studies showed that PD is characterized by increased brain temperature that depends on the net balance between the heat released by the mitochondrial respiratory chain, the cerebral blood flow, and core body temperature [47,48]. Although increased brain temperature may play a key role in neurodegeneration, its relationship with mitochondrial dysfunction and the clinical features is still not clear [47,48]. The integrated use of functional ^31^P-MRS, ^1^H-MRS, and arterial spin labelling (for the study of brain perfusion) will allow one to break new ground in the study of PD pathogenesis.

## Figures and Tables

**Table 1:** Demographic and clinical characteristics of the ^31^phosphorus magnetic resonance spectroscopy (P-MRS) studies included in the review (alphabetical order).

Authors	Participants^a^	Age at PD Onset^b^	Disease Duration	PD Clinical Scales^c^	Medications	^31^P-MRS	Area of Interest	Main Results^d^
	(Male/Femal)	(average)	(average)			Design		
Barbiroli et al. [24]	13 PD (8/5)	55.2 ± 10 s.d.	11.7 ± 4.9 s.d.	H&Y	L-dopa (13 PD)	Resting-state	Occipital Lobes	Pi: PD > HC
	15 MSA (12/3)					(1.5T)		PCr: MSA < PD
	16 HC (N.A.)							
Brockmann et al. [25]	13 GBA-PD (10/3)	49.5 y.o. (from 28 to 65)	5.5 (from 3 to 12)	UK Brain Bank Criteria	N.A.	Resting-state	Putamen	
	19 HC (11/8)			H&Y		(3T)	Midbrain	GPE: GBA-PD > HC
				UPDRS				
Hattingen et al. [26]	29 PD (23/6)	N.A.	N.A.	UK Brain Bank Criteria	L-dopa (23/23)	Resting-state	Putamen	ATP: PD < HC
	19 HC (9/10)			H&Y	Dopamine Agonists (7/23)	(3T)	Midbrain	PCr: PD < HC
				UPDRS				
Hilker et al. [22]	2 PD PINK1+ HZ (0/2)	N.A.	11.5 ± 0.7 s.d.	H&Y	L-dopa (2 PD PINK1 HZ)	Resting-state	Putamen	βATP; PCr: PINK1+ HZ > PINK1- DZ, HC
	9 PD PINK1- DZ (7/2)			UPDRS		(3T)		GPC; GPE: PINK1+ HZ > PINK1- DZ, HC
	23 HC (6/17)							
Hu et al. [27]	10 PD (N.A.)	N.A.	5.9 ± 3.8 s.d.	UK Brain Bank Criteria	L-dopa (10/10)	Resting-state	Temporoparietal Cortex	Bilateral Temporoparietal:
	9 HC (N.A.)			H&Y	Dopamine Agonists (4/10)	(1.5T)	Occipital Cortex	Pi/βATP: PD > HC
							Thalamus	Right Temporoparietal:
							Pallidus	Pi: PD > HC
							Midbrain	Thalamus, Pallidus, Midbrain:
								βATP: PD < HC
								PME/βATP; PDE/βATP; PCr/βATP:
								PD > HC
Montagna et al. [28]	10 PD (7/3)	55.6 ± 7.3 s.d.	6.8 ± 4.7 s.d.	H&Y	L-dopa (10/10)	Resting-state	Frontal Lobes	Pi: PD > HC
	9 HC (9/0)					(1.5T)	Basal Grey structures	
Rango et al. [23]	20 PD (10/10)	N.A.	7 ± 2.5 s.d.	UK Brain Bank Criteria	L-dopa (20/20)	Functional	Visual Cortex	PCr + βATP (Recovery): PD < HC
	20 HC (10/10)			H&Y	Dopamine Agonists (6/20)	(1.5T)		
Rango et al. [29]	1 PD PINK1 DZ (0/1)	46	16	UPDRS	L-dopa (10/10)	Functional	Visual Cortex	PCr + βATP (rest): EOPD < PD; HC
	10 PD (0/10)	N.A.	N.A.		L-dopa + Dopamine Agonist (PD PINK1)	(1.5T)		PCr + βATP (activation): EOPD < PD; HC
	10 HC (0/10)							PCr + βATP (recovery): EOPD < PD; HC
Weiduschat et al. [30]	20 PD (10;10)	N.A.	N.A.	UK Brain Bank Criteria	L-dopa (4M;2F)	Resting-state	Striatum	HEP: Male PD < Female PD
					Dopamine Agonists (2M;4F)	(3T)	Temporoparietal GM	
	12 HC (7;5)			UPDRS				
Weiduschat et al. [31]	20 PD (10;10)	55.6 ± 12.0 s.d.	3.2 ± 1.8 s.d.	UK Brain Bank Criteria	L-dopa (3/10)	Resting-state	Striatum	No energetics difference
	15 HC			UPDRS	L-dopa + Dopamine agonists (3/10)	(3T)	Temporoparietal GM	between control and PD at
				H&Y	Dopa agonists (5/10)			Early Stage

^a^ PD: Idiopathic Parkinson’s disease; MSA: Multiple system atrophy; HC: Healthy controls; GBA-PD: Parkinson’s disease with *GBA* gene mutation; PD-PINK1 +: Parkinson’s disease with *PINK1* gene mutation with severe symptoms; PD-PINK1-: Parkinson’s disease with *PINK1* gene mutation with mild symptoms; HZ: Homozygous; DZ: Dizygous; ^b^ s.d.: Standard deviation; N.A: Not available; ^c^: H&Y: Hoehn and Yahr scale; UK Brain Bank Criteria: UK Parkinson’s Disease Society Brain Bank Diagnostic Criteria; UPDRS: Unified Parkinson’s Disease Rating Scale; ^d^: Pi: Inorganic phosphate; PCr: Phosphocreatine; Cr: Creatine; GPE: Glycerophosphoethanolamine; GPC: Glycerophosphocholine; ATP: Adenosine triphosphate; PME: Phosphomonoester; PDE: Phosphodiester; HEP: High-energy phosphates.

## References

[1] Kalia L., Lang A.E. (2015). Parkinson’s disease. Lancet.

[2] Ferguson L.W., Rajput A.H., Rajput A. (2015). Early-onset vs. Late-onset Parkinson’s disease: A Clinical-pathological Study. Can. J. Neurol. Sci..

[3] Gustavsson E.K., Trinh J., McKenzie M., Bortnick S., Petersen M.S., Farrer M.J., Aasly J.O. (2017). Genetic Identification in Early Onset Parkinsonism among Norwegian Patients. Mov. Disord. Clin. Pract..

[4] Park J.S., Davis R.L., Sue C.M. (2018). Mitochondrial dysfunction in Parkinson’s disease: New Mechanistic Insights and Therapeutic Perspectives. Curr. Neurol. Neurosci. Rep..

[5] Chen C., Turnbull D.M., Reeve A.K. (2019). Mitochondrial dysfunction in Parkinson’s disease—Cause or consequence?. Biology.

[6] Nandipati S., Litvan I. (2016). Environmental exposures and Parkinson’s disease. Int. J. Environ. Res. Public Health.

[7] Bury A.G., Pyle A., Elson J.L., Greaves L., Morris C.M., Hudson G., Pienaar I.S. (2017). Mitochondrial DNA changes in pedunculopontine cholinergic neurons in Parkinson disease. Ann. Neurol..

[8] Chu Y., Goldman J.G., Kelly L., He Y., Waliczek T., Kordower J.H. (2014). Abnormal alpha-synuclein reduces nigral voltage-dependent anion channel 1 in sporadic and experimental Parkinson’s disease. Neurobiol. Dis..

[9] Reeve A.K., Park T.K., Jaros E., Campbell G.R., Lax N.Z., Hepplewhite P.D., Krishnan K.J., Elson J.L., Morris C.M., McKeith I.G. (2012). Relationship between mitochondria and α-synuclein: A study of single substantia nigra neurons. Arch. Neurol..

[10] Lill C.M. (2016). Genetics of Parkinson’s disease. Mol. Cell. Probes.

[11] Scarffe L.A., Stevens D.A., Dawson V.L., Dawson T.M. (2014). Parkin and PINK1: Much more than mitophagy. Trends Neurosci..

[12] Valente E.M., Abou-Sleiman P.M., Caputo V., Muqit M.M.K., Harvey K., Gispert S., Ali Z., Del Turco D., Bentivoglio A.R., Healy D.G. (2004). Molecular Pathways of Neurodegeneration in Parkinson’s Disease. Science.

[13] Pickrell A.M., Youle R.J. (2015). The roles of PINK1, Parkin, and mitochondrial fidelity in Parkinson’s disease. Neuron.

[14] Geisler S., Holmström K.M., Treis A., Skujat D., Weber S.S., Fiesel F.C., Kahle P.J., Springer W. (2010). The PINK1/Parkin-mediated mitophagy is compromised by PD-associated mutations. Autophagy.

[15] Kostic M., Ludtmann M.H.R., Bading H., Hershfinkel M., Steer E., Chu C.T., Abramov A.Y., Sekler I. (2015). PKA Phosphorylation of NCLX Reverses Mitochondrial Calcium Overload and Depolarization, Promoting Survival of PINK1-Deficient Dopaminergic Neurons. Cell Rep..

[16] Amo T., Saiki S., Sawayama T., Sato S., Hattori N. (2014). Detailed analysis of mitochondrial respiratory chain defects caused by loss of PINK1. Neurosci. Lett..

[17] Gan-Or Z., Amshalom I., Kilarski L.L., Bar-Shira A., Gana-Weisz M., Mirelman A., Marder K., Bressman S., Giladi N., Orr-Urtreger A. (2015). Differential effects of severe vs mild GBA mutations on Parkinson disease. Neurology.

[18] Gegg M.E., Schapira A.H.V. (2018). The role of glucocerebrosidase in Parkinson disease pathogenesis. FEBS J..

[19] Li H., Ham A., Ma T.C., Kuo S.H., Kanter E., Kim D., Ko H.S., Quan Y., Sardi S.P., Li A. (2019). Mitochondrial dysfunction and mitophagy defect triggered by heterozygous GBA mutations. Autophagy.

[20] Henchcliffe C., Shungu D.C., Mao X., Huang C., Nirenberg M.J., Jenkins B.G., Beal M.F. (2008). Multinuclear Magnetic Resonance Spectroscopy for in Vivo Assessment of Mitochondrial Dysfunction in Parkinson’s Disease. Ann. N. Y. Acad. Sci..

[21] Ross B., Bluml S. (2001). Magnetic resonance spectroscopy of the human brain. Anat. Rec..

[22] Hilker R., Pilatus U., Eggers C., Hagenah J., Roggendorf J., Baudrexel S., Klein J.C., Neumaier B., Fink G.R., Steinmetz H. (2012). The Bioenergetic Status Relates to Dopamine Neuron Loss in Familial PD with PINK1 Mutations. PLoS ONE.

[23] Rango M., Bonifati C., Bresolin N. (2006). Parkinson’s disease and Brain Mitochondrial Dysfunction: A Functional Phosphorus Magnetic Resonance Spectroscopy Study. J. Cereb. Blood Flow Metab..

[24] Barbiroli B., Martinelli P., Patuelli A., Lodi R., Iotti S., Cortelli P., Montagna P. (1999). Phosphorus magnetic resonance spectroscopy in multiple system atrophy and Parkinson’s disease. Mov. Disord..

[25] Brockmann K., Hilker R., Pilatus U., Baudrexel S., Srulijes K., Magerkurth J., Hauser A.-K., Schulte C., Csoti I., Merten C.D. (2012). GBA-associated PD. Neurodegeneration, altered membrane metabolism, and lack of energy failure. Neurology.

[26] Hattingen E., Magerkurth J., Pilatus U., Mozer A., Seifried C., Steinmetz H., Zanella F., Hilker R. (2009). Phosphorus and proton magnetic resonance spectroscopy demonstrates mitochondrial dysfunction in early and advanced Parkinson’s disease. Brain.

[27] Hu M.T.M., Taylor-Robinson S.D., Chaudhuri K.R., Bell J.D., Labbé C., Cunningham V.J., Koepp M.J., Hammers A., Morris R.G., Turjanski N. (2000). Cortical dysfunction in non-demented Parkinson’s disease patients. A combined 31P-MRS and 18FDG-PET study. Brain.

[28] Montagna P., Pierangeli G., Cortelli P., Zaniol P., Funicello R., Lugaresi E., Barbiroli B. (1993). Brain oxidative metabolism in Parkinson’s disease studied by phosphorus 31 magnetic resonance spectroscopy. J. Neuroimaging.

[29] Rango M., Arighi A., Marotta G., Ronchi D., Bresolin N. (2013). PINK1 parkinsonism and Parkinson disease: Distinguishable brain mitochondrial function and metabolomics. Mitochondrion.

[30] Weiduschat N., Mao X., Beal M.F., Nirenberg M.J., Shungu D.C., Henchcliffe C. (2014). Sex differences in cerebral energy metabolism in Parkinson’s disease: A phosphorus magnetic resonance spectroscopic imaging study. Park. Relat. Disord..

[31] Weiduschat N., Mao X., Beal M.F., Nirenberg M.J., Shungu D.C., Henchcliffe C. (2015). Usefulness of Proton and Phosphorus MR Spectroscopic Imaging for Early Diagnosis of Parkinson’s Disease. J. Neuroimaging.

[32] Masuda T., Dobson G.P., Veech R.L. (1990). The Gibbs-Donnan near-equilibrium system of heart. J. Biol. Chem..

[33] Hu M.T., Taylor-Robinson S.D., Chaudhuri K.R., Bell J.D., Morris R.G., Clough C., Brooks D.J., Turjanski N. (1999). Evidence for cortical dysfunction in clinically non-demented patients with Parkinson’s disease: A proton MR spectroscopy study. J. Neurol. Neurosurg. Psychiatry.

[34] Ullah M.F., Ahmad A., Bhat S.H., Abu-Duhier F.M., Barreto G.E., Ashraf G.M. (2019). Impact of sex differences and gender specificity on behavioral characteristics and pathophysiology of neurodegenerative disorders. Neurosci. Biobehav. Rev..

[35] Nilsen J., Brinton R.D. (2003). Mechanism of estrogen-mediated neuroprotection: Regulation of mitochondrial calcium and Bcl-2 expression. Proc. Natl. Acad. Sci. USA.

[36] Cereda E., Barichella M., Cassani E., Caccialanza R., Pezzoli G. (2013). Reproductive factors and clinical features of Parkinson’s disease. Park. Relat. Disord..

[37] Papa S., Sardanelli A.M., Capitanio N., Piccoli C. (2009). Mitochondrial respiratory dysfunction and mutations in mitochondrial DNA in PINK1 familial Parkinsonism. J. Bioenerg. Biomembr..

[38] Prebil M., Jensen J., Zorec R., Kreft M. (2011). Astrocytes and energy metabolism. Arch. Physiol. Biochem..

[39] Lin M.T., Beal M.F. (2006). Mitochondrial dysfunction and oxidative stress in neurodegenerative diseases. Nature.

[40] O’Regan G., Desouza R.M., Balestrino R., Schapira A.H. (2017). Glucocerebrosidase Mutations in Parkinson Disease. J. Parkinsons. Dis..

[41] Mazzulli J.R., Xu Y.H., Sun Y., Knight A.L., McLean P.J., Caldwell G.A., Sidransky E., Grabowski G.A., Krainc D. (2011). Gaucher disease glucocerebrosidase and α-synuclein form a bidirectional pathogenic loop in synucleinopathies. Cell.

[42] Endo T., Sakaue H. (2019). Multifaceted roles of porin in mitochondrial protein and lipid transport. Biochem. Soc. Trans..

[43] Mori A., Hatano T., Inoshita T., Shiba-Fukushima K., Koinuma T., Meng H., Kubo S.I., Spratt S., Cui C., Yamashita C. (2019). Parkinson’s disease-associated iPLA2-VIA/PLA2G6 regulates neuronal functions and α-synuclein stability through membrane remodeling. Proc. Natl. Acad. Sci. USA.

[44] Zadali R., Ghareghozloo E.R., Ramezani M., Hassani V., Rafiei Y., Chiyaneh S.M., Meratan A.A. (2019). Interactions with and Membrane Permeabilization of Brain Mitochondria by Amyloid Fibrils. J. Vis. Exp..

[45] Rango M., Bozzali M., Prelle A., Scarlato G., Bresolin N. (2001). Brain activation in normal subjects and in patients affected by mitochondrial disease without clinical central nervous system involvement: A phosphorus magnetic resonance spectroscopy study. J. Cereb. Blood Flow Metab..

[46] Fox P., Raichle M., Mintun M., Dence C. (1988). Nonoxidative glucose consumption during focal physiologic neural activity. Science.

[47] Rango M., Piatti M., Di Fonzo A., Ardolino G., Airaghi L., Biondetti P., Bresolin N. (2016). Abnormal brain temperature in early-onset Parkinson’s disease. Mov. Disord..

[48] Rango M., Arighi A., Bonifati C., Bresolin N. (2012). Increased brain temperature in Parkinson’s disease. Neuroreport.

